# What's it gonna take? Lessons learned for youth-friendly mental health services research

**DOI:** 10.3389/frhs.2025.1623179

**Published:** 2025-12-11

**Authors:** Chiachen Cheng, Hafsa B. Siddiqui, Arianne St. Jacques, Sumit Kumar, Kyle Vader, Madyson Campbell, Sabrina Maisonneuve, Elizabeth Minnery

**Affiliations:** 1 Section of Psychiatry, Clinical Sciences Division, Northern Ontario School of Medicine (NOSM) University, Thunder Bay, ON, Canada; 2 Dr. Gilles Arcand Centre for Health Equity, NOSM University, Thunder Bay, ON, Canada; 3 Independent Researcher, Thunder Bay, ON, Canada; 4 Undergraduate Medical Education, NOSM University, Sudbury, ON, Canada; 5 Undergraduate Medical Education, NOSM University, Thunder Bay, ON, Canada; 6 Independent Researcher, North Bay, ON, Canada

**Keywords:** youth engagement, mental health services, service evaluation, youth service users, youth participatory action research, integrated youth services

## Abstract

**Introduction:**

Over half of the children and youth with mental illness do not receive appropriate or adequate treatment in both Canada and the United States. The burden of mental illness and substance use is the leading cause of disability due to years lost to disability, leading to the youth mental health crisis. Despite ongoing efforts to improve mental health and substance use services, many youth disengage prematurely, with evidence that this leads to poorer outcomes. In this paper, we explore the question: how to use youth-friendly methods in research for service improvement.

**Methods:**

We used innovative and participatory action mixed methods. Youth between the ages of 12 and 25, with lived experience accessing mental health and addiction services, were recruited for focus groups. The focus groups were stratified based on their level of service needs, and data were analyzed using thematic analysis. The themes were interpreted into a fictional narrative summarized in an animated video. This video was embedded in a survey that was sent to the participants. The purpose was to validate the analysis and explore the factors that led them to participate. A descriptive analysis of the quantitative data and an inductive content analysis of the qualitative data were completed for the survey.

**Results:**

A total of 44 youth completed the screening to stratify the level of need. Fourteen youth participated in three pilot focus groups, and another 24 participated in four focus groups stratified by need. The mean age was 22.3 years, and 78% and 22% identified as male and female, respectively. Youth-friendly research was the main theme, with two main sub-themes: youth want to participate in research, and there were strategies for research approaches involving youth service users. Fundamentally, choice throughout the process was important.

**Conclusion:**

Youth service users want to be engaged meaningfully. Youth are not afraid to speak their truth and want opportunities to provide their unique perspectives. Service improvements from youth service-user feedback may lead to improved outcomes with full treatment because youth remain engaged with services. Service improvement may need youth-friendly research.

## Introduction

According to the World Health Organization (WHO), mental illness and substance use represent one of the most disabling health burdens among youth, ranking as the leading cause of years lost to disability among individuals aged 10–24 years (1, 2). In Canada, an estimated 1.2 million children and youth are affected by mental illness, yet fewer than 20% receive appropriate treatment (3). Similarly, in the United States prior to the COVID-19 pandemic, half of the 7.7 million children and youth with mental health needs did not receive adequate treatment, and the pandemic has amplified this need (4). The disparity between need and the long-term impact of unmet needs forces us to critically examine what services can do to meet these unmet needs.

Youth health service research has focused on improving youth engagement in services with the aspiration of improving health outcomes. In their 2002 policy document *Adolescent Friendly Health Services: An Agenda for Change* (5), the WHO argued that improving the “friendliness” of adolescent health services would lead to increased access to services (by decreasing barriers to access) and decreased long-term burden of disease through early access and engagement with health services. Since then, researchers (6–8) have expanded this call to action to also include youth mental health and substance use services. Some of the researchers who answered the WHO call to action also examined youth-friendly services in integrated youth services (IYS) (9–11)

One research strategy to improve youth engagement with services is to evaluate services using Youth Participatory Action Research (YPAR). YPAR is a research methodology under the broad area of community participatory research. YPAR actively engages youth in research processes where youth participation (as participants and/or researchers) leads to advocacy and change. YPAR principles include collaboration, empowerment, subjectivity, deconstruction, and transformation (12–15). Researchers use YPAR methods because youth have a unique perspective on services and how services can be improved.

Despite this push to improve youth engagement with mental health services, a 2013 meta-analysis found that between 28% and 75% of youth prematurely disengage from mental health services (16). Youth who prematurely disengage from treatment are significantly more likely to have worse outcomes, including persistent mental health issues, academic difficulties, substance use, and delinquency (16–20). Clinicians are often unaware of why youth are dissatisfied with their services and how this contributes to youth prematurely disengaging (21). Clinicians who demonstrate respect and interest in their patients' satisfaction with services are more likely to have youth stay engaged with their treatment (22).

The Mental Health and Addictions Youth Network (MAYNet) (2022–2025) project sought to understand how integrated youth services/centers can provide youth-friendly services to youth with a diverse range of needs. The goal was to understand how to meet the needs of youth with “high risk” needs and “low risk” needs in the same IYS.

We found that youth who participate in research may not be the same as youth with lived experience accessing or receiving care (i.e., youth service users) and that youth service users may have very different perspectives and different challenges than those who participate in research. YPAR and other strategies have focused on youth engagement in the co-design of mental health services (23). Yet the life circumstances of these two groups may be different, and the strategies needed to understand the perspectives of these two groups may also be different.

While research has focused on how to engage youth *in general*, the literature lacks clear guidance about best practices for operationalizing research (for service improvement) involving youth receiving the services (24–27). To explore this gap, we will use innovative mixed methods to explore the sub-question of what the best practices are for *youth-friendly* methods in *research* for service improvement.

## Methods

In this paper, we consider service research to be under the umbrella of program evaluation. We are researching the implementation of quality improvement initiatives for mental health and substance use services. While the distinction is not necessarily relevant for this paper, the study was designed from this perspective (28).

### Study design

We used YPAR principles and mixed methods (29) with focus groups followed by an online survey to engage people with lived experience accessing youth mental health and addiction services.

### Ethical approval

This study was approved by Lakehead University's Research Ethics Board (REB) in Thunder Bay, Ontario, Canada. We followed standard research practices as outlined by TCPS2 (Tri-Council Policy Statement for Ethical Conduct for Research Involving Humans) (30) to ensure the integrity of the data collection, analysis, and confidentiality throughout the research process. As part of the REB approval, we had a protocol for screening and obtaining consent from youth participants and data collection and analysis. Youth researchers participated in study design, data collection, analysis (HS), and manuscript preparation (HS, MC, and SM).

### Reflexivity

We approached this research inductively and within a constructivist worldview, whereby we acknowledged that multiple realities can simultaneously exist within the context of our research question, while also being particularly attuned to “real-world” and practical applications (29). Our research team consisted of two child and adolescent psychiatrists (CC, AS), a PhD candidate in psychology (HS), a facilitator from a district public health unit (SK), three undergraduate medical students (KV, MC, SM), and a researcher with lived experience accessing and receiving mental health services (EM).

### Participants and recruitment

Youth between the ages of 12–25 years were recruited from across Canada using Reddit and X (formerly Twitter). The inclusion criteria were as follows: (i) have currently or previously accessed mental health and/or substance use services; (ii) can read, write, and speak in English, and (iii) can use technology to participate in virtual focus groups. Therefore, the recruitment post specified youth under 25 years who have currently, or previously, accessed mental health and/or substance use services.

After potential participants reached out by social media or email, they were asked to fill out a Qualtrics (31) questionnaire with their contact information. After registration, each participant was invited to a screening call with a research assistant who ensured capacity to consent to the study and then proceeded with the screening questions to assess their level of service needs (see Supplemental File 1: Screening call questions).

Based on their answers to the screening questions, participants were assigned a “needs score” to indicate whether they had high, medium, or low levels of needs. Answering “yes” to questions 3, 5, 6, or 11 resulted in 2 points, while answering yes to the other questions was assigned 1 point; the maximum score was 15. *High risk* was conceptualized by a need for more intensive services to mitigate risks such as suicidality, legal charges, and incarceration (e.g., service needs such as access to safe injection sites, addressing a history of childhood maltreatment, and/or involvement of child protection). *Low risk* was operationalized as less need for services that mitigate risk, and instead focused on supportive services (e.g., resume assistance and employment support). Not all participants who were screened were selected for focus groups; youth were selected to participate in focus groups based on their needs score, with a focus on youth with low and high risk needs.

Every participant who completed the screening process was entered into a draw to win a $100 gift card. Youth who completed the screening were invited to participate in a focus group based on the level of need stratification. Those who completed the focus group also received a $25 gift card.

### Data collection

Between October 2022 and January 2023, focus group questions were pilot tested in three online focus groups via Zoom (32). All youth (i.e., high, medium, and low needs youth) who reached out were invited to participate. In consultation with an author (EM)/researcher with lived experience, data from these pilot groups were used to adjust the focus group questions. Adjustments included collecting demographic data, specifically to clarify how we were defining stigma, and an added question about how youth want to participate in research about service improvement (e.g., question 4 in Supplementary File 2. The guiding questions with probes that the focus group facilitator(s) (HS, AS) used are in Supplementary File 2.

Between September 2023 and December 2024, a second round of recruitment was completed using the same social media approach. This time, youth participants were purposively sampled and stratified into focus groups based on their screening score (e.g., level of risk based on their level of need). From this group of youth who completed the screening, four focus groups were completed online, with two consisting of low to medium needs youth, and the other two groups consisting of high needs youth. Focus groups were recorded, transcribed verbatim, anonymized, and stored on a secure university drive to ensure participant confidentiality.

After analysis (as described below) of the focus group data, we constructed a fictional narrative based on our inductive thematic analysis. Using Vyond (33), this narrative was produced into an animated video (Supplementary Video from YouTube). In the video, following the presentation of this narrative, we ended the video by summarizing the themes that we inducted from our data. The video link was embedded in an online survey. The purpose of this survey was to validate our analysis of the themes that were presented in the video and to further explore the factors that led the youth to participate in research about service improvement. The online survey was created using Qualtrics (31) and emailed to all youth (both focus group participants and youth who were screened but did not participate in the focus groups). Youth who completed the survey received a $15 online gift card. See Supplementary File 3 for survey questions.

### Data analysis

For the focus group data, we used Braun and Clarke's (34) approach to inductive thematic analysis. Two authors (HS and SK) independently inductively coded the transcripts, searching for patterns. There was no limit to codes nor a specific focus of coding other than a curiosity about patterns for low-risk and high-risk youth; our analysis was constructionist because we were more focused on the social and cultural context of the data, and not necessarily individual motivation for the youth participant answers. HS and SK met with the principal investigator (CC) to discuss the codes, and with the latent level (e.g., generating a hypothesis) of analysis, we developed global themes. For the survey data, the collected data were verified to ensure only participants who had completed the initial screening process answered the survey, regardless of whether they were selected for a focus group. A descriptive analysis of the quantitative data was conducted, and we completed an inductive content analysis (35) of the short-answer qualitative responses. The findings from the focus groups and online survey were integrated through a triangulation process, whereby themes identified in the qualitative focus group data were compared with patterns from the survey responses to generate a more robust understanding of participant perspectives.

### Rigor

To establish rigor and build trustworthiness of the data, we engaged in regular reflexive dialog as a research team about themes and have included representative quotations to illustrate our findings (36).

## Results

### Demographics

A total of 161 individuals answered the social media posts. Of these, we were able to contact and book screening calls with 44 youth. All 44 participants completed the screening process and agreed to participate in focus groups. A total of three pilot focus groups involving 14 youth participants (August to December 2022) and four focus groups involving 24 youth participants (November to December 2023) were completed online. Only the focus groups in 2023 were stratified into groups according to our “needs score.” Of the four focus groups in 2023, groups 1 and 4 were the two low/medium groups with average scores of 5 and 3.4, respectively. Groups 2 and 3’s average scores were 8.5 and 9, respectively (the remaining six youth who participated in screening were “medium” needs level, and were not invited to participate in focus groups).

Demographics were collected only from the 24 youth from the 2023 cohort. Across all four focus groups, there were 3 who lived in Northern Territories (Yukon, Nunavut, and Northwest Territories), 7 from the Western provinces (British Columbia, Alberta, Saskatchewan, Manitoba), 24 from Central Canada (Ontario, Quebec), and 4 from the Atlantic provinces (Prince Edward Island, New Brunswick, Nova Scotia, Newfoundland and Labrador). The mean age of the participants was 22.3 years (17–25 years). Table 1 summarizes their self-identified ethnicity and other demographics.

**Table 1:** Demographics of Focus Group Participants.

**Mean Age**	22.3 yrs (17–25 yrs)					
**Gender**	**Male**			**Female**		
	78.3 % (*n*=18)			21.7% (*n*=5)		
**Ethnicity**	**Mixed**	**Black**	**White/ European**	**First Nations**	**Middle Eastern**	**Latino**
	39.1% (*n*=9)	17.4% (*n*=4)	13.0% (n=3)	8.7% (*n*=2)	8.7% (*n*=2)	4.3% (*n*=1)
**Education**	**University certificate/ diploma/degree**		**College CEGEP**	**Trade Certificate**		**High School & Equivalent**
	73.9% (*n*=17)		8.7% (*n*=2)	4.4% (*n*=1)		13.0% (*n*=3)
**Orientation**	**Heterosexual**			**2SLGBTQI+**		
	65.2% (*n*=15)			34.8% (*n*=8)		

CEGEP, Collège d'enseignement général et professionnel.

For the validation survey, 49 youth responded. Of these responses, 22 were removed because they were not from one of the 44 participants who completed the screening process in 2023. Of the remaining 27 youth who completed the survey, 3 participated only with the screening process, and 24 were part of the focus groups.

### Analysis

In our inductive analysis, youth-friendly research was the main theme. Two global themes about youth-friendly research also emerged: youth want to participate in research, and youth had suggestions about how to implement research involving youth. The results about service improvements are being prepared in a separate manuscript. This paper focuses on the *youth-friendly research* themes that emerged from our study.

### An opportunity to be heard

In the focus groups, youth appreciated the opportunity to participate in research, including the initial screening about their level of need, and the focus groups. Participants were clear that they wanted to be engaged and wanted to be part of the research (and evaluation) process, especially with the goal of service improvement.

Of the youth who answered the validation survey, 55% had previously had the opportunity to participate in a Youth Advisory Group (YAG). The youth resonated with Jane's story (from the validation video) about being too busy to participate in a YAG but still wanting to give feedback. Moreover, 81% of youth expressed that participating in research is important to them because they want a platform for their opinions to be heard. To them, participating in research provides an opportunity to give feedback that researchers can use to influence real change by advocating through decision-makers and other researchers.

In addition to the opportunity to be heard by service researchers and decision-makers, youth also enjoyed the opportunity to hear and learn from the experiences of other youth and perhaps not feel so isolated and alone.

“I didn't know how the virtual session would actually look like. So personally, I have learnt a lot and I had hopes of learning from other people's experience. So I believe I have achieved my main purpose for being here because taking part today, I have been able to listen to other participants. And to know that, okay, I’m not just the only person having this kind of thoughts or this kind of experience.” (Participant from the high-risk focus group).

#### Implementing youth-friendly research

Youth participants had a lot to say about how to create youth-friendly opportunities for them to feel heard. Not only do they need to feel that they have autonomy in whether they provide feedback, but they also need to feel that they can give frank and honest feedback without repercussions on their care. It mattered to them *how* and with *whom* they gave feedback.

“I feel focus groups of this sort should be better used, because, you know, in a setting where there's … a member in the call that is clearly … an official, or like, clearly older …  holding a call, or like someone that you know you can rely on like the older person.” (Participant from the high-risk focus group).

A research process (from recruitment to data collection) that provides choice was fundamentally important to youth. To this end, a mixed-methods approach to data collection gives youth a choice about *how* to participate with researchers. Some preferred surveys, while others preferred focus groups; yet others preferred that surveys be administered after interviews/focus groups to provide some anonymity to their responses. They suggested that to strike a balance between individual preferences is to follow up focus groups with open-ended surveys where participants can add additional comments they may not have felt comfortable voicing during the interview/group session. A mixed-methods approach to data collection offers youth the opportunity to engage with researchers in a manner that is comfortable for them.

“Everyone has different choices, different decisions, preferences … [some] feel comfortable filling out random surveys, while others might prefer an interview or focus group. But for me I feel what matters is that individual[s] are given the opportunity to choose from this variety of choices.” (Participant from the high-risk group).

Furthermore, individualized recruitment engaged high needs youth to participate. As shown in Supplementary File 1, the screening questions were very personal. Despite this, the youth described the process as “comfortable” and one that “fostered and encouraged” inclusion. In fact, there were no differences between how high and low needs' participants felt about the screening. Meeting a member of the research team, one-on-one, gave them the opportunity to anticipate the types of questions they may be asked after recruitment, in the data collection process.

High needs youth indicated a preference for being placed in groups with youth who are similar to their own lived experience. Although participants frequently mentioned that they enjoyed listening to diverse viewpoints, for sensitive topics (such as what they need from mental health and/or substance use services), they prefer to be with youth who are similar to them.

“One of the factors that that made me sure was I think the fact that I think was the screening. We are told that we actually will be placed, based on the similarities of I don't know … So all the factors that will actually influence me choosing would be the fact that people that were having discussions are people that have similar experiences with. (Participant from the high-risk group).

## Discussion

The MAYNet goals were to understand how to improve mental health and substance use services to better meet the needs of youth and ultimately improve youth mental health outcomes. We were interested in how IYS can be improved to meet the diverse range of needs and whether high needs youth perspectives may be different from youth with low level of needs. While answering this question, we found that to improve services adequately, youth services need to conduct evaluative research that is authentic to their clients' perspectives. We found that to engage youth in service improvement, we need to adopt youth-friendly research practices. This concept has also been suggested by the practical recommendations to clinicians provided by Hawke et al. (24) who made recommendations about ways in which we can guide a youth-friendly approach to youth engagement in research. The lessons from MAYNet may have service policy implications that may be broader than IYS.

### Youth want to be engaged *meaningfully*

MAYNet participants were clear that they wanted to engage and wanted to be part of the research process for service improvement. While only 27% of the 161 youth who initially responded were reachable for screening bookings, all 44 youth who were booked for screening also consented to participate in focus groups. Youth wanted to give feedback, and in fact, they were willing to disclose personal details about themselves (e.g., the screening process) in a one-on-one format, where the format was private, and they were given the opportunity to ask questions.

McCabe et al. (25) have previously described the importance of creating a space for open discussion to effectively engage youth in mental health services research. This is similar to our findings that for meaningful, authentic engagement with youth, they need to feel confident in their research contributions. High level of need youth told us that confidence is built by ensuring the environment in which they provide feedback is comfortable by grouping them with peers with similar challenges and social, ethic, and economic contexts. We found that by ensuring their comfort and reassuring them that their voices will be used to inform change, youth can overcome their apprehension (about the risk of disclosure) to contribute to research and service improvement.

Youth want to engage in service research because they want to help drive change. Youth want the opportunity to be heard by their peers and by decision-makers. This desire to drive change was a recurring theme in the MAYNet results: youth expressed this theme in the focus groups, and they validated this finding in the survey.

### Choice

It is not necessarily a new finding that youth value choice. We found that a mixed-methods, multimodal research design offers an optimal choice for youth to participate in research. In our study, we used individual consent and screening, focus groups (stratified based on level of need), and a follow-up survey (with embedded video illustrating the thematic analysis of the results in a fictional narrative). This intensive process likely reduced our participation numbers; yet we were surprised that the youth who participated not only were comfortable with the screening process, but they felt included and expressed that they enjoyed the experience, including the focus groups.

It is possible that meeting with a member of the research team one-on-one provided the youth with the opportunity to better anticipate the types of questions they may be asked during the focus groups, and they retained autonomy of their level of participation. Furthermore, the multimodal data collection also provided the flexibility that the youth may have needed, especially if they were not as comfortable with one research method over the other. This approach is consistent with the literature about upholding the principle of choice for youth by offering multiple avenues for participation, which aligns with principles of youth-friendliness that prioritize autonomy, accessibility, and individual preference (37). Moreover, the rapid review by Jones et al. (38) similarly underscored the importance of prioritizing flexibility and adaptability when asking youth experts about their lived experience to promote meaningful engagement of youth in mental health service improvement.

YPAR methods often engage youth by having a youth advisory group/board (YAG) advising the project at all stages. However, a recent systematic review identified that in addition to youth who engage in YPAR initiatives, other youth service users also want to be involved in shaping the care they receive; however, the literature lacks clear guidance about how to operationalize youth service-user feedback (39). Only ∼50% of the youth who completed the validation survey in our study had participated in YAGs previously, and many agreed that “life happens,” and they did not have the time to fully participate in YPAR activities, including YAG. Our data support that youth service users are not necessarily the youth who are participating in YPAR methods. This mirrors findings from Campbell et al. (40) who found that certain populations of youth prefer less intense levels of research engagement and participation.

Health service researchers are accustomed to adapting to shifting priorities or contexts. When working with youth, it is fundamental to remain agile and flexible. Using multimodal data collection methods, engaging youth service users meaningfully and specifically, and being prepared to pivot according to youth needs offer an optimal choice.

### Limitations of the study

The global pandemic required that the research protocol be shifted to online platforms. While online platforms had advantages such as reaching a broad youth population across the country and allowed for some anonymity when the camera was turned off, we recognize that social media recruitment and online data collection also have their limitations (41). Since our study was conducted online, one of the inclusion criteria was that the youth must have access to the internet and a device that could support videoconferencing. As a result, we were not able to include youth who are unable to use these technologies.

We recognize that the generalizability of our findings is limited by the geographic context in which the study was conducted and that our sample was mental health and substance use service users who predominantly accessed “traditional” models of care, which may limit the extent to which these results can be applied to broader youth populations or diverse service contexts. When considering our findings, it is important to acknowledge that the participants included in this research were from a Canadian context and limited to youth with prior experience accessing mental health and/or substance use services. As such, the transferability of our findings to other geographical regions and to practice areas beyond youth mental health and substance use is not clear. Finally, while the results of this research reflect the views of participants in this research, including youth with lived experience accessing mental health and/or substance use services across diverse regions of Canada, the findings may not be representative of all youth who access these services, especially for those youth without access to digital technologies or with differing service use experiences or preferences.

## Conclusions

Mental health and substance use services need to do better to address the youth mental health crisis. Youth service users want to help drive the change. Services are also striving for ways to improve mental health and substance use outcomes in youth. Therefore, involving youth with lived experience as key partners in service evaluation offers an opportunity for mutual benefit. Overall, youth who are meaningfully engaged in service improvement evaluation experience feelings of empowerment and increased self-esteem (38). Authentic service improvement may need youth-friendly research to obtain the necessary feedback from youth.

Some of the lessons learned have important service policy implications, especially in IYS contexts. Youth want to give feedback. They want the opportunity to be heard by both their peers and by decision-makers, and they are interested in driving change within the services they access. Key principles of youth engagement in service evaluation include prioritizing flexibility in recruitment, consent, and data collection by using strategies such as creating comfortable, individualized spaces and ensuring youth know their voices are meaningful (24). Giving them a sense of control over whom and how they share their feedback will also foster meaningful engagement. Service decision-makers need to be willing to hear their feedback, even if contentious, and youth need to feel confident in their research contributions. Further, service decision-makers must have confidence in the credibility of knowledge provided by youth with lived experience for youth participation to influence service improvement (38). As part of our commitment to translating these findings, a series of knowledge exchange workshops was held with stakeholders, to be discussed in future papers.

There are many opportunities for future directions, such as questions that explore more barrier-free research opportunities for youth service users to participate in research. We need to shift how service improvement feedback is solicited and be inclusive of youth who may experience barriers by finding multimodal data collection methods to accommodate different levels of engagement with the research. The possibilities are vast and exciting to consider the potential for evidence-informed excellence; these findings may be relevant not only to IYS but also to other youth mental health and substance use services. By using youth-friendly research approaches, we can help address the youth mental health crisis in a way that is inclusive and empowering for youth.

## Data Availability

Anonymized raw data supporting the conclusions may be available by the authors until 2032.
