# Supplementary material for: What's it gonna take? Lessons learned for youth-friendly mental health services research
Source: Front Health Serv. 2025 Dec 11;5:1623179. doi: 10.3389/frhs.2025.1623179 (PMC12738366; doi:10.3389/frhs.2025.1623179)
Supplement: Supplementary file 1 [file Supplementaryfile1.docx]

| **Questions about substance use** | |
| --- | --- |
| 1. Could you please tell us how often you use either drugs or alcohol? | a. Once a week or more  b. Once a month or more  c. Less than once a month  d. Never |
| 2. Have you ever tried to stop using alcohol or drugs, but been unable to? | Yes  No |
| **Questions about difficulties with the law** | |
| 3. Have you ever been charged with anything? | Yes  No |
| 4. Are you close to individuals who have had conflicts with the law? | Yes  No |
| 5. Has one of your parents or siblings ever gone to prison, or been in or out of jail? | Yes  No |
| **Questions about your relationship with your family, now and when you were a kid** | |
| 6. Are you currently living, or have previously lived in Foster Care? | Yes  No |
| 7. Would you say that you have had a father figure when growing up? (Even if it wasn’t your biological father) | Yes  No |
| 8. Do you feel that you have a support person who you can trust and who you feel connected to? | Yes  No |
| 9. Have you ever been in a situation where you had trouble finding enough money to pay for food? | Yes  No |
| **Questions are about your experiences with your mental health** | |
| 10. Have you ever stayed overnight in a hospital unit or crisis center for mental health or substance use issues (not including the emergency room)? | Yes  No |
| 11. Have you ever attempted suicide? | Yes  No |

**Supplemental 1 : Screening Call Questionnaire**
