# Supplementary material for: What's it gonna take? Lessons learned for youth-friendly mental health services research
Source: Front Health Serv. 2025 Dec 11;5:1623179. doi: 10.3389/frhs.2025.1623179 (PMC12738366; doi:10.3389/frhs.2025.1623179)
Supplement: Supplementary file 2 [file Supplementaryfile2.docx]

**Supplemental 2: Focus Group Guiding Questions**

1. Previous research into the idea of youth-friendly mental health and addiction services has defined youth-friendly as:

“A youth-friendly service is one that is accessible, appealing, flexible, confidential and integrated, where youth feel respected, valued, and welcome to express themselves authentically, without discrimination of any kind; ”

*Possible Probes*

- What parts of this definition stand out to you as the most important? Is anything hard to understand, or feeling like it doesn’t relate to you?

- Imagine someone who has different life experience and different needs from your own. Do you think there are ways for both your needs and the needs of someone very different from you to be met by the same service?

- What is the difference between a service that is youth-friendly, and one that is child-friendly or adult-friendly?

2. The purpose of Integrated Youth Services is to act as a one-stop-shop for youth living with mild to severe mental health conditions, and who come from a variety of backgrounds. With such a diverse group of people accessing the service, what effect, if any, do you think stigma will have on a diverse group of service users and their desire to continue engaging with the service?

*Possible Probes*

- Tell us about your experiences with stigma in accessing youth or mental health services

- What kind of stigmas or prejudices do you have about other people with mental health or substance use issues? Where do you think those may have come from?

- How do you think stigma towards others would affect your experience in accessing mental health or addictions services?

- How do you think stigma towards yourself would affect your experience in accessing mental health or addictions services?

- Do you think that bringing together people from different backgrounds or experiences helps to erase stigma, or does it make it worse?

3. We want the results of this study to help teach the people who design youth services to make them friendlier to all users. Knowing the barriers that you have faced in the past and everything that we have talked about today, tell us what the ideal service would look like for you?

*Possible probes:*

- If you have ever gone to a place that says it’s youth friendly but didn’t feel friendly to you, what was it that made you feel that way?

- Would a one-stop-shop that also offers hands on skill building and leadership workshops be something you look for in a mental health and substance use service? Why or why not?

- What exactly is it which an integrated youth service can offer which you believe would be most beneficial for someone looking for help with mental health and/or substance use issues?

- If you had your choice of services, what are some things that you would look for in a service that you would want to access, and what might turn you off of a service?

- If you have accessed services during the pandemic, have there been any experiences that have been really positive or negative with that?

- How do you feel about accessing services through your school, doctor’s office, and other places that are already familiar to you? What about places that are less familiar?

4. In order to learn more about whether new types of services, such as IYS, are effective, researchers like us need to collect information from clients. Sometimes that takes the form of interviews or focus groups like this one, and often it’s through surveys or questionnaires that you fill out before or after your appointments (e.g. in the waiting room or online at home). How do you feel about taking part in this type of research?

*Possible probes:*

- Do you have preferences of one type of research style over another? Why?

- What are some ways that researchers could make this process easier or more “youth friendly”?

- Have you experienced this type of data collection with mental health and/or addiction services? Did you feel it was mandatory or were you given a choice whether to take part?

- What factors would make you more or less likely to take part in this type of research?
