# Supplementary material for: What's it gonna take? Lessons learned for youth-friendly mental health services research
Source: Front Health Serv. 2025 Dec 11;5:1623179. doi: 10.3389/frhs.2025.1623179 (PMC12738366; doi:10.3389/frhs.2025.1623179)
Supplement: Supplementary file 3 [file Supplementaryfile3.docx]

**Supplemental 4: Validation Survey**

| In our project, we met with everyone, but only some people went on to complete focus groups. Were you part of our focus groups?  *Yes No* |
| --- |
| Have you ever been involved or had the opportunity to become involved in a youth advisory group?  *Yes No* |
| Based on your experience in the youth advisory group, please rate the following statements:  *Strongly disagree Disagree Neither Agree/nor disagree Agree Strongly agree*   - It was held at a convenient time - It was easy to participate (e.g. accessible transportation - I felt my contribution was important - I felt understood |
| Your contribution at the screening stage was very important. Please rate the following statements:  *Strongly disagree Disagree Neither Agree/nor disagree Agree Strongly agree*   - I was very disappointed about not doing focus groups - As long as I got to participate in some way, it didn't matter if I did the focus groups - I felt heard |
| Do you have any feedback on the screening process? What should we keep doing, and what can we change for the better? |
| In the video, Jane was assigned into a focus group with people who are similar to her. When talking about sensitive topics, do you want to be placed with youth who have similar backgrounds to you, or would you prefer to be in groups with diverse people?  *Similar Diverse Doesn't matter* |
| After you’ve shared your thoughts with researchers, what would you like them to do with this information? Check all that apply.   - *Advocate on behalf of youth to policymakers* - *Publish in academic journals where other researchers and service providers can read the findings* - *Create spaces for youth participants to share their experiences with a broader audience* - *Other* |
| Why was it important for you to participate in this study? Check all that apply.   - *The gift card* - *Offering my opinion* - *Seemed interesting* - *I wanted to be heard* - *Other* |
| Would you be comfortable with someone from within the service collecting feedback from you, or would you prefer someone from outside the service?   - *Within the service* - *Outside the service* - *Doesn't matter* |
| In the video, Jane was assigned into a focus group with people who are similar to her. When talking about sensitive topics, do you want to be placed with youth who have similar backgrounds to you, or would you prefer to be in groups with diverse people?  *Similar Diverse Doesn't matter* |
| After you’ve shared your thoughts with researchers, what would you like them to do with this information? Check all that apply.   - *Advocate on behalf of youth to policymakers* - *Publish in academic journals where other researchers and service providers can read the findings* - *Create spaces for youth participants to share their experiences with a broader audience* - *Other* |
| Why was it important for you to participate in this study? Check all that apply.   - *The gift card* - *Offering my opinion* - *Seemed interesting* - *I wanted to be heard* - *Other* |
| Would you be comfortable with someone from within the service collecting feedback from you, or would you prefer someone from outside the service?  *Within the service Outside the service Doesn't matter* |
| Please rate each statement  *Strongly disagree Disagree Neither Agree/nor disagree Agree Strongly agree*   - Regardless of who is collecting feedback, I’m not concerned that my care will be impacted - Regardless of who is collecting feedback, I’m comfortable being honest with my feedback. - I’m concerned that my care will be impacted by the feedback I give. - I feel I can be more honest giving feedback to someone from outside the service - *I would be more comfortable giving my feedback to someone outside the service* |
